# The CRTh2 polymorphism rs533116 G > A associates with asthma severity in older females

**DOI:** 10.3389/fmed.2022.970495

**Published:** 2022-10-13

**Authors:** Nami Shrestha Palikhe, Constance A. Mackenzie, Christopher Licskai, Richard B. Kim, Harissios Vliagoftis, Lisa Cameron

**Affiliations:** ^1^Division of Pulmonary Medicine, Department of Medicine and Alberta Respiratory Centre, University of Alberta, Edmonton, AB, Canada; ^2^Division of Respirology, Department of Medicine, Western University, London, ON, Canada; ^3^Division of Clinical Pharmacology, Department of Medicine, Western University, London, ON, Canada; ^4^Division of Clinical Pharmacology and Toxicology, Ontario Poison Centre, The Hospital for Sick Children, Toronto, ON, Canada; ^5^Department of Pathology and Laboratory Medicine, Schulich School of Medicine and Dentistry, Western University, London, ON, Canada

**Keywords:** CRTh2, asthma severity, polymorphism, female, sex difference, type 2 inflammation, genetic variation

## Abstract

**Background:**

CRTh2 is G protein coupled receptor for prostaglandin D2 (PGD)_2_ expressed by immune cells that drive type 2 inflammation such as CD4^+^ T cells (Th2), eosinophils and group 2 innate lymphoid cells (ILC2) as well as structural cells including smooth muscle and epithelium. CRTh2-expressing cells are increased in the blood and airways of asthmatics and severe asthma is characterized by increased activity of the PGD_2_-CRTh2 pathway. The *CRTh2* single nucleotide polymorphism (SNP) rs533116 G > A is associated with development of asthma and increased Th2 cell differentiation.

**Objective:**

To examine whether CRTh2 rs533116G > A associates with asthma severity. Since severe asthma is more common in females than males, we performed a sex-stratified analysis.

**Methods:**

Clinical data from asthmatics (*n* = 170) were obtained from clinic visits and chart review. Asthma severity was assessed according to ERS/ATS guidelines. Peripheral blood cells were characterized by flow cytometry and qRT-PCR. Genotyping was performed by TaqMan assay.

**Results:**

Older females (≥45 years) homozygous for minor A allele of rs533116 were more likely to have severe asthma, lower FEV_1_, a higher prescribed dose of inhaled corticosteroid and more type 2 inflammation than females carrying GA or GG genotypes. Comparing females and males with the AA genotype also revealed that women had more type 2 inflammation.

**Conclusions and significance:**

The polymorphism CRTh2 rs533116 G > A associates with severe asthma and type 2 inflammation in older females. This study reveals a gene-sex-aging interaction influencing the effect of CRTh2 on asthma severity.

## Introduction

Asthma is a heterogeneous disease with many endotypes. Type 2 high asthma is the most common form and is mediated by the cytokines IL-4, IL-5, and IL-13 (1). These cytokines are produced primarily by Th2 (T-helper 2) cells and ILC2s (group 2 innate lymphoid cells) and together promote type 2 inflammation and infiltration of other important inflammatory cells such as eosinophils and basophils (2–4). Th2 cells (5–7) and ILC2 (4) are defined by the expression of CRTh2 (chemoattractant homologous receptor expressed by Th2 cells). Other inflammatory cells including eosinophils and basophils and structural cells such as smooth muscle and epithelium also express CRTh2 (8–10).

While both Th2 cells and ILC2 produce type 2 cytokines, these cells are considered to play inherently different roles in immune responses. Th2 cells are differentiated and activated by allergen-primed dendritic cells (11) and are memory T cells that circulate between the lymph nodes and periphery providing surveillance and allergen specific responses (12, 13). ILC2 can be found in the blood, but at 1,000-fold lower numbers than Th2 cells (14); this is considered to be due to their role as innate cells residing primarily at mucosal sites. ILC2 are activated by IL-25 and IL-33, cytokines released from the airway epithelium following exposure to allergens (15) or other environmental factors such as cigarette smoke or viral and/or bacterial infections [reviewed in (16)]. Both Th2 cells and ILC2 have been shown to be increased in the blood and airways of severe compared to mild/moderate asthmatics (14, 17, 18). CRTh2 is a G protein coupled receptor for prostaglandin D_2_ (PGD)_2_ (19, 20) and activation of CRTh2 mediates chemotaxis (19) as well as type 2 cytokine expression (21). Since PGD_2_ is produced by both allergen-activated mast cells (22) and microbial-activated macrophages (23), the PGD_2_-CRTh2 pathway is considered to play a role in contributing to both day to day asthma symptoms as well as exacerbations [reviewed in (24, 25)].

Genetic variations in CRTh2 have been associated with development of asthma and other allergic phenotypes (26, 27). We reported that the minor allele of rs533116 G > A, within a *CRTh2* enhancer region, was associated with higher levels of CRTh2 expression on circulating CD4^+^ T cells and eosinophils and *in vitro* differentiated Th2 cells (28). We recently observed that the proportion of CD4^+^CRTh2^+^ T cells (Th2 cells) are increased in severe asthma (17). Others have shown more CRTh2-expressing cells and higher PGD_2_ levels in bronchial alveolar lavage and epithelial brushings from severe asthmatics (18). To date, no studies have investigated whether this polymorphism is associated with clinical characteristics of asthma including severity of the disease.

In this study, we investigated whether the genotype status of rs533116 is associated with severe asthma and/or indices of asthma severity. In light of our emerging understanding of the importance of sex differences in health and disease, coupled with reports that females are more likely to suffer from severe asthma and to have more severe symptoms (29), we additionally performed a sex-stratified analysis. We found that the homogeneous minor allele of rs533116 (AA genotype) associated with asthma severity and degree of type 2 inflammation exclusively in females. Collectively, our results suggest that genetic variation in CRTh2 may play a sex-specific role in asthma severity.

## Materials and methods

### Subjects

The institutional ethics review boards of the University of Alberta and Western University approved this study. Patients were recruited and consented from the tertiary care Asthma Clinics at the University of Alberta, Edmonton Alberta and London Health Sciences Centre/St. Joseph’s Health Care, London, Ontario. Inclusion criteria were age >18 years and a physician diagnosis of asthma. Severe asthma was defined as asthma that requires treatment with high dose inhaled corticosteroids plus a second controller (and/or systemic corticosteroids) to prevent it from becoming “uncontrolled” or that remains “uncontrolled” despite this therapy. Inadequate symptom control was defined by any one of the following: (i) airflow limitation (FEV_1_ < 80% predicted after bronchodilator medication withheld); (ii) Asthma Control Questionnaire (ACQ) > 1.5; (iii) frequent severe exacerbations (two or more systemic corticosteroids bursts (>3 days each); (iv) serious exacerbation (at least one hospitalization or ICU stay in the previous year) (30).

### Genotyping

Using the Wizard^®^ Genomic DNA Purification Kit (Promega, Madison, WI, USA), DNA was isolated from peripheral blood mononuclear cells or whole blood following manufacturer instructions. *CRTh2* rs533116 [g. −6,391 bp (G > A), upstream of the translation start site] genotyping was performed using TaqMan^®^ allelic discrimination assay (Applied Biosystems, Foster City, CA, USA).

### Quantitative real time polymerase chain reaction

Whole blood (2 ml) was collected in PAX gene tubes and total RNA was isolated using the PAXgene Blood RNA Kit (PreAnalytiX, Qiagen, BD, Mississauga, ON, Canada) according to the manufacturer’s instruction. Reverse transcription (RT) reactions were performed using 1 μg of RNA, oligo-dT primers and Superscript II Reverse Transcriptase (Invitrogen, Burlington, ON, Canada). TaqMan gene expression assays for *CRTh2* (Hs00173717_ml) and *GATA3* (Hs00231122_ml) were quantified using pre-designed Taqman assays (Applied Biosystems, Carlsbad, CA, USA), 19 μl of TaqMan gene expression master mix and 1 μl of cDNA. *GAPDH* mRNA was used as an internal control (housekeeping control) and was quantified by a custom ^6^FAM-labeled TAMRA probe (5′-AAA TCC CAT CAC CAT CTT CCA GGA GCG A-3′) (Applied Biosystems) and the following primers *GAPDH*-F (5′-CAA GGCT GAG AAC GGG AAG-3′) and *GAPDH*-R (5′-GCA AAT GAG CCC CAG CCT T-3′). The PCR protocol consisted of 10 min at 95°C followed by 40 cycles of 30 s at 95°C and 60 s at 60°C. All samples were run in triplicate. Differences in Ct values of the gene of interest and the house keeping gene *GAPDH* were used to calculate delta Ct (ΔCt). Relative fold changes (RFC) were then calculated using the 2^–ΔΔ^*^Ct^* algorithm. All ΔCt were subtracted from one subjects’ ΔCt (lowest Ct value) to calculate ΔΔCt and fold increase then calculated using the ΔΔCt as a negative exponent to the base of 2 (2^–ΔΔ^*^Ct^*).

### Flow cytometry

Determining the proportion of circulating cells expressing CRTh2 was performed as we described previously (17, 31). Briefly, whole peripheral blood was stained using antibodies against CD4, CCR3, CRTh2, or isotype control and fixed with paraformaldehyde (2%). Flow cytometry analysis was performed using a BD LSR II Flow Cytometer, with gates set in accordance with the profiles of the isotype control and/or negative control beads. The proportion of Th2 cells was determine as the proportion of CD4^+^CRTh2^+^ cells/peripheral white blood cells (pWBC). CRTh2^+^eosinophils were identified as the proportion of high side scatter (SSc*^high^*) cells expressing CCR3 and CRTh2/WBC. Results were analyzed using FlowJo^®^ (TreeStar, Ashland OR, USA).

### Statistical analyses

Difference in the mean value of phenotypic clinical characteristics was compared using the Mann-Whitney *U*-test or independent *t*-test (based on normality test) for continuous variables and Fisher exact *t*-test for categorical variables. Significant departures of genotype frequency from Hardy-Weinberg equilibrium were determined by Fisher exact *t*-test. For associations of the CRTh2 rs533116 polymorphism (dominant and recessive model) with severe asthma, odds ratios were calculated by binary logistic regression adjusted for sex, age and/or BMI according to analysis and *p*-values determined by Chi-Square. Statistical analyses were performed using SPSS (version 28, Chicago, IL, USA). Statistical significance was set at *P* < 0.05.

## Results

### Asthma population

Table 1 represents the clinical and demographic data of the population with physician diagnosed asthmatics (*n* = 170) recruited from specialty asthma clinics, separated into patients with severe or mild/moderate asthma determined by ERS/ATS guidelines (30). There were no differences in age, BMI or sex distribution between the two groups. Severe asthmatics had lower forced expiratory volume in one second (FEV_1;%_ predicted), ratio of FEV_1_/forced vital capacity (FVC) and were taking higher doses of inhaled corticosteroids (ICS). Those in the severe asthma group were more likely to have FEV_1_ < 80% predicted and to have needed oral corticosteroids, indicating poorer symptom control (Table 1). A sex-stratified analysis showed that males had lower FEV_1_ and FEV_1_/FVC than females and in older subjects (>45 years) that males were more likely to require oral corticosteroids (39%) than females (17%, *p* = 0.014; Supplementary Table 1).

**TABLE 1:** Clinical characteristics of study subjects.

Characteristics	Total (*N* = 170)	Mild/moderate (*N* = 109)	Severe (*N* = 61)	*p*
Age	52.0 ± 1.2	53.0 ± 1.5	50.3 ± 1.9	0.296
Sex (% female)	106 (62.4)	73 (67.0)	33 (54.1)	0.097
BMI	31.1 ± 0.6	31.4 ± 0.8	30.5 ± 0.9	0.538
FEV_1_ (% predicted)	79.7 ± 20.2	82.2 ± 19.1	75.1 ± 21.5	**0.027**
FVC (% predicted)	93.0 ± 17.1	93.1 ± 16.1	92.7 ± 19.0	0.872
FEV_1_/FVC	65.9 ± 0.9	67.8 ± 1.2	62.4 ± 1.7	**0.004**
Total daily ICS	1091.6 ± 53.2	712.5 ± 31.4	1769.1 ± 84.1	**<0.0001**
Smoking status **No (%)	79 (46.5)	49 (45.0)	30 (50.0)	0.565
**Criteria to determine inadequate asthma control**				
FEV_1_ (<80% predicted), No (%)	84 (49.4)	47 (43)	37 (61)	**0.029**
OCS usage, No (%)	28 (16.5)	5 (4.6)	23 (37.7)	**<0.001**
ACQ score (No)	1.72 (136)	1.64 (89)	1.86 (47)	0.218
Urgent care visit/hospitalization last year, No (%)	16 (23.9)	8 (20)	8 (29.6)	0.225

FEV_1_, Forced expiratory volume in 1 s; FVC, Forced vital capacity; ICS, Inhaled Corticosteroid, budesonide equivalent; Smoking status ever **Total: *n* = 168; Mild/moderate: *n* = 108; Severe: *n* = 60; No, Number of subjects. Bold values are to indicate significance.

### CRTh2 rs533116 G > A is associated with severe asthma in older females

**FIGURE 1:**
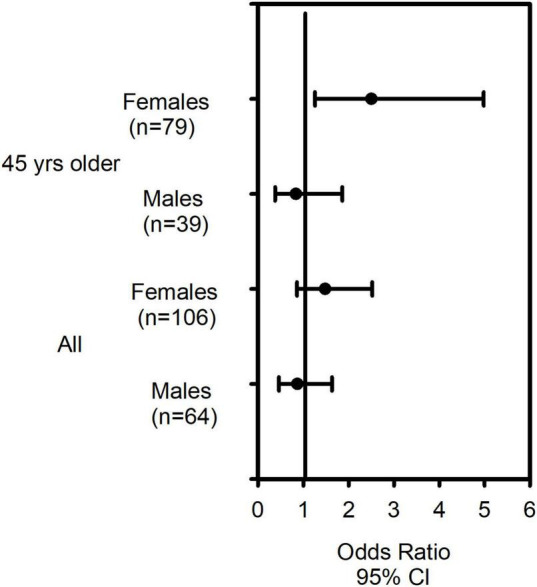
The CRTh2 rs533116 G > A polymorphism increases risk of severe asthma in older females. Asthmatics with mild/moderate (*n* = 109) and severe asthma (*n* = 61) were genotyped and odds ratio (OR) and 95% CI were determined. Females 45 years and older carrying the AA genotype were at increased risk of having severe asthma compared to those of GG/GA genotype. Analysis using binary logistic regression were adjusted for age, sex and BMI.

We previously reported CRTh2 rs533116 G > A to be associated with susceptibility to develop asthma (28, 32). In this study, after confirming that genotype frequencies within this asthma cohort exhibited Hardy Weinberg equilibrium (Supplementary Table 2), we assessed the relationship between this polymorphism and diagnosis of severe asthma. Examining the entire population, we found no significant associations with severe asthma, although those homozygous for minor A allele (AA genotype) showed a trend for significance in females (OR = 1.48 [95% CI = 0.86–2.52]), but not males (OR = 0.87 [95% CI = 0.46–1.63]; Supplementary Table 3). Recently, the probability of having severe asthma was shown to increase with each year of life until age 45 (33). We therefore restricted our further analyses to older asthmatics. In females 45 years or older, the AA genotype was significantly associated with having severe asthma (OR = 2.50 [95% CI = 1.26–4.98], *p* = 0.009), while no association was observed in males (OR = 0.84 [95% CI = 0.38–1.86]; *p* = 0.660; Supplementary Table 4 and Figure 1).

### What clinical parameters of severity are influenced by CRTh2 rs533116 G > A?

**FIGURE 2:**
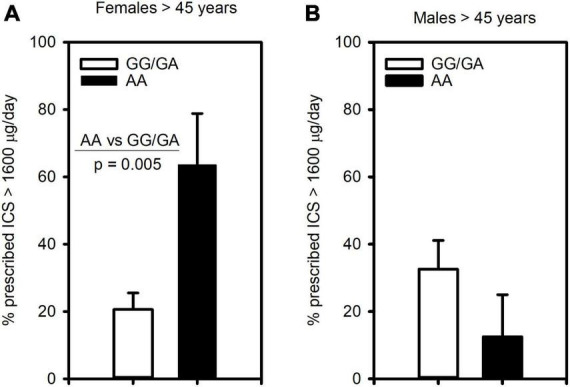
The CRTh2 rs533116 G > A polymorphism is associated with ICS usage. Asthmatics were genotyped and assessed for the likelihood of being prescribed high dose ICS (>1600 μg/day, budesonide equivalent), an indicator of asthma severity. **(A)** Older females (AA = 11, GG/GA = 68) and **(B)** older males (AA = 8, GG/GA = 31) were assessed for the influence of the A allele on this outcome. Statistical differences determined Mann-Whitney *U*-test.

Since this polymorphism was associated with severe asthma, we next assessed its relationship to clinical outcomes of asthma severity. Examining ICS usage, we found similarly to severe asthma, that females 45 years and older homozygous for the A allele (AA genotype) were significantly more likely to be prescribed more than 1,600 μg/day of ICS (budesonide equivalent) than those of GG/GA genotype (Figure 2A). In contrast, this association was not observed in males (Figure 2B).

**FIGURE 3:**
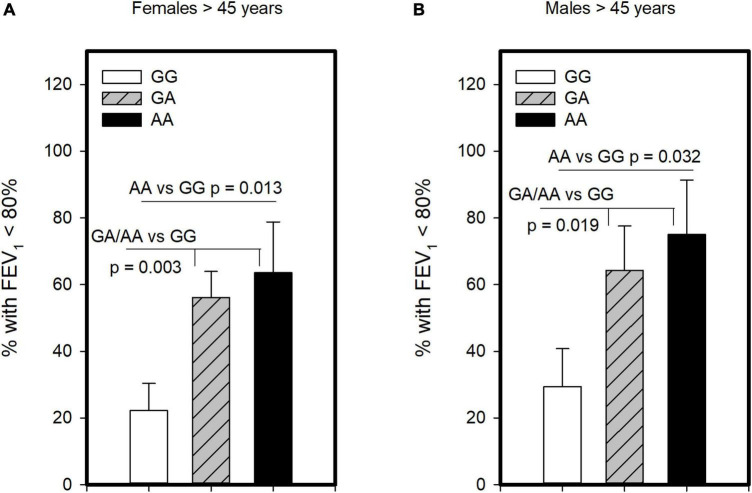
The CRTh2 rs533116 G > A polymorphism is associated with lung function. Asthmatics were genotyped and assessed for the likelihood of having FEV_1_ below the normal range of 80%, an indicator of asthma severity. **(A)** Older females (AA = 11, GA = 41, GG = 27) and **(B)** older males (AA = 8, GA = 14, GG = 17) were assessed for the influence of A allele on this outcome. Statistical differences determined Mann-Whitney *U*-test.

Another critical determinant of asthma severity is lung function. Unlike analysis for severe asthma and ICS usage, we found no evidence that homozygosity for A allele (AA genotype) was associated with having lower FEV_1_ compared to those with GG/GA. However, examining those with GG and AA genotypes showed significantly lower FEV_1_ in the combined population 45 years and older (*p* = 0.005) as well as older females (*p* = 0.023), though not males. As such, we examined this polymorphism under the dominant model and found that older asthmatics with GA or AA genotype had FEV_1_ levels lower than GG in the combined group (*p* = 0.001), females (*p* = 0.004) and males (*p* = 0.036; Supplementary Table 5). We also examined association of this polymorphism with symptom control, assessing its association with having FEV_1_ below the normal range. This analysis revealed, with the dominant and genotypic models, that older females (Figure 3A) and older males (Figure 3B) carrying the A allele were more likely to have FEV_1_ < 80% than those with the GG. As such, these results suggest both GA and AA genotypes influence FEV_1_. Other measures of symptom control were only available in subjects recruited from the University of Alberta (*n* = 59, Supplementary Table 6) and showed no significant differences across genotypes. Although there was a promising trend for CRTh2 rs533116 AA to coincide with increased likelihood of exacerbation in females (Supplementary Figure 1), suggesting this should be studied further. Only a few subjects (*n* = 5) needed > 3 cycles of oral corticosteroid/year. Collectively, the nature by which CRTh2 rs533116 G > A associates with asthma severity appears to depend on biological sex and age.

### Does CRTh2 rs533116G > A influence type 2 inflammation?

**FIGURE 4:**
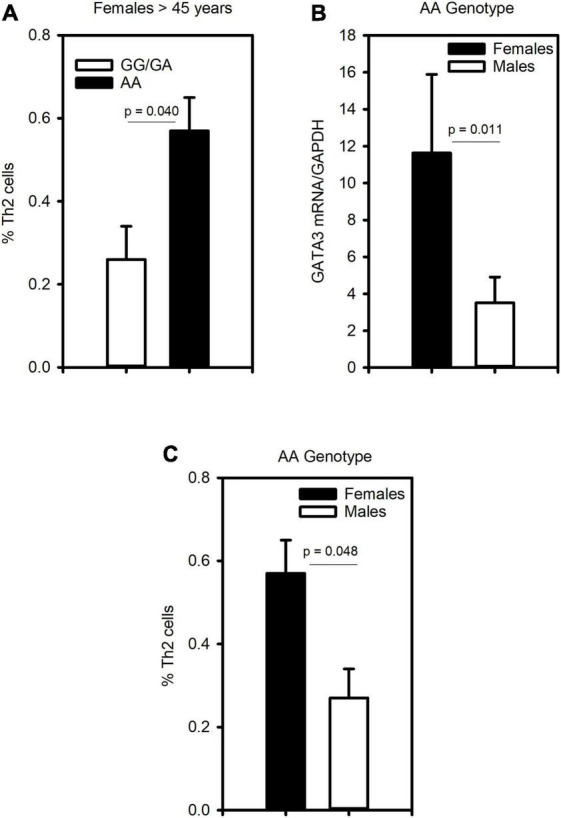
The CRTh2 rs533116 G > A polymorphism is associated with increased type 2 inflammation. A subset of asthmatics were characterized for type 2 inflammation. **(A)** Influence of the AA genotype on the proportion of Th2 cells (CD4^+^CRTh2^+^/peripheral white blood cells) determined by flow cytometry in older females (45 years and older; AA = 3, GG/GA = 13). **(B)** Level of GATA3 mRNA from whole blood and **(C)** proportion of Th2 cells in older females (*n* = 3) and males (*n* = 3) carrying the AA genotype. Statistical differences determined Mann-Whitney *U*-test.

To assess whether CRTh2 rs533116G > A influences the degree of type 2 inflammation, the subset recruited from the University of Alberta were extensively characterized (*n* = 59; Supplementary Table 6). This analysis showed that females of AA genotype had more CD4^+^ T cells, eosinophils, *GATA3* and *CRTh2* mRNA than those carrying GA/GG genotype, but only a trend for elevated levels of circulating Th2 cells. No significant differences in type 2 inflammation were observed across genotypes in males (Table 2). These findings were similar even when those on oral corticosteroids were removed from the analysis (females, *n* = 1; males, *n* = 4; data not shown). Since a gradual increase in type 2 inflammation with age has been reported (34), we specifically examined older asthmatics. This analysis revealed that in older females carrying the AA genotype coincided with having significantly more Th2 cells (Figure 4A) compared to those females with GG or GA genotypes. Moreover, in older asthmatics the level of *GATA3* mRNA (Figure 4B) and proportion of Th2 cells (Figure 4C) were higher in females than males of AA genotype. These results suggest this polymorphism enhances type 2 inflammation in females, but not males, and that the effect is more evident with age.

**TABLE 2:** Association of CRTh2 rs533116 G > A with type 2 inflammation in all asthmatics.

	CD4^+^ T cells	*Eos	CRTh2 mRNA	GATA3 mRNA	Th2 cells
**Female**					
GG/GA (*n* = 25)	6.65 ± 1.13	1.47 ± 0.29	4.20 ± 0.74	3.42 ± 0.47	0.29 ± 0.04
AA (*n* = 6)	12.76 ± 1.53^$^	3.20 ± 0.36^$^	8.50 ± 2.35^$^	8.57 ± 2.39	0.46 ± 0.07^$^
*P*	0.007	0.003	0.022	0.019	0.061
**Male**					
GG/GA (*n* = 20)	6.59 ± 0.86	1.83 ± 0.57	4.69 ± 0.87	3.72 ± 0.67	0.26 ± 0.03
AA (*n* = 7)	6.75 ± 1.43	1.33 ± 0.36	3.72 ± 0.78	4.47 ± 0.83	0.21 ± 0.04
*p*	ns	ns	ns	ns	ns

*Eos, eosinophils; *p*: Mann-Whitney Rank U-test; ^$^*p* < 0.05, females vs. males with AA.

## Discussion

Development of chronic inflammatory diseases involve gene-by-environment interactions (35). Although many studies have focused on external environmental exposures, such as air pollution (36), the internal environment generated by biological sex is perhaps the most fundamental and complex of interacting factors. Males and females have different gene complements that shape the internal environment and this sex-specific genetic architecture contributes to human disease (37). Age is another factor potentially influencing the impact of genetic variation, with physiologic changes occurring throughout the lifespan (38). The prevalence of asthma is known to differ with age and sex. In childhood, higher prevalence is observed in males than in females, while asthma risk increases in girls after puberty (39). This shift is attributed to internal changes in hormonal milieu (40). By adulthood, females are twice as likely as males to have a severe asthma diagnosis and severe asthma symptoms (29, 41).

Despite the plethora of studies examining genetics of asthma and the progress toward personalized medicine (42), sex-specific analyses are often not considered. Mersha et al. showed the importance of stratifying by sex as 55% of the genetic variants identified in sex-specific analyses were not found in the combined analysis and effect sizes were often larger (43). Though some sex-specific risk alleles for asthma (44), asthma-related quantitative traits IgE and FEV_1_ (45) and regulatory pathways have now been identified (46, 47), a deeper understanding is still needed. Further, age-related changes influencing asthma risk and asthma severity throughout life may also exhibit sex-specific effects. Lung function, for instance, peaks ∼20 years (48) and then declines with age (49). Type 2 inflammation was recently shown to increase with age, particularly in the fourth decade (34).

Our study revealed a gene-sex-aging relationship between CRTh2 533116 G > A and various parameters related to asthma severity. Indeed, we found that in older females the AA genotype was associated with having a diagnosis of severe asthma, increased likelihood of taking high dose ICS and having FEV_1_ (% predicted) below the normal range (<80%). Many aspects of type 2 inflammation were also increased in females, but not males with the AA genotype, though the proportion of Th2 cells was only significantly higher in those older than 45 years. The mechanism underlying why the AA genotype associates with increased type 2 inflammation only in females remains unclear but we hypothesize that this may be related to their stronger propensity to develop type 2 responses (50–52). Estrogen has been shown to induce IL-4 and IL-13 expression in murine models of asthma (53, 54), while serum estradiol during the luteal phase was positively correlated with airway levels of IL-5 (55). In males, the AA genotype did not influence risk of having severe asthma, coincide with higher ICS usage or type 2 inflammation, which in turn may be due to androgen levels; androgens have been shown to suppress type 2 inflammation (50–52). In our study males were more likely to be taking systemic corticosteroids, which could blunt detection of type 2 responses. Removing these subjects from the analysis, however, gave similar results and so it seems unlikely the lack of association of AA with increased type 2 inflammation in males is due to corticosteroid usage. Although peripheral detection of type 2 inflammation (blood eosinophil counts) has become the standard for determining eligibility for anti-type 2 medications (56), airway assessment is considered more accurate (57). As such, additional studies examining association of CRTh2 rs533116 AA and airway levels of CRTh2^+^ cells in males and females are still needed to fully understand its influence on type 2 inflammation. Intriguingly, there may be sex differences in the role of peripheral vs. lung cell contribution to type 2 inflammation, since androgens directly reduced ILC2-mediated (50, 51), but indirectly reduced Th2 cell-mediated type 2 inflammation (52).

In terms of the influence of the CRTh2 rs533116 G > A polymorphism on FEV_1_, the combined analysis of males and females showed a trend for significance for the AA genotype to influence risk for lower FEV_1_ (recessive model, *p* = 0.055) but, in contrast to diagnosis of severe asthma, no effect was observed in sex-stratified analysis. Instead, we observed a graded gene dose effect of 1 and 2 copies of the A allele (GA and AA genotypes) on FEV_1_. A number of studies now report preference for assessing genetic association using a genotypic model as they can be more sensitive than the additive model, even when there is an intermediate heterozygote effect (58, 59). Association between the FTO (Fat mass and obesity-associated) genetic variant rs1421085 and severe obesity, for example, was higher using a comparison of homozygous genotypes than the additive model (60). Using this approach, we found the AA genotype was associated with increased likelihood of having FEV_1_ below the normal range (<80%) than those of GG genotype, in both females and males. Taken together, our results suggest the influence of CRTh2 rs533116 G > A on asthma severity emerges in females as they age, when declining lung function (49) interacts with genotype- and/or age-mediated increases in type 2 inflammation (34). The fact that in males this polymorphism associates with lower lung function, but not type 2 inflammation and asthma severity, suggests the potential for sex differences in asthma etiology. Moreover, it highlights the importance of future work assessing whether CRTh2 rs533116 G > A directly influences lung function *per se* and its impact on other lung conditions.

PGD_2_ release from mast cells serves as a chemotactic factor drawing CRTh2-expressing inflammatory cells to allergen-exposed tissues (8, 22, 61, 62). On Th2 cells, PGD_2_ activation of CRTh2 mediates production of IL-4, IL-5 and IL-13, amplifying the type 2 response (21). Our previous study showed association of CRTh2 rs533116 AA with higher eosinophil and T cell expression of CRTh2 in young adults with self-reported asthma (28). Here we report this association persists in physician diagnosed asthma. Whole genome discovery of regulatory regions identified H3K4me1 binding to the CRTh2 rs533116 locus, indicating this polymorphism resides within an enhancer region [HaploReg^1^; (63)]. Transcription factor binding site analysis also revealed that the CRTh2 rs533116 G allele (but not A allele) contains a putative NFAT site. Mechanistically, this could result in increased CRTh2 expression since we previously showed this transcription factor reduces CRTh2 transcription (64). If so, CRTh2 rs533116AA could drive unfettered transcription resulting in high level CRTh2 mRNA and more CRTh2-expressing cells.

Differences in the influence of this polymorphism on type 2 inflammation and FEV_1_ could be the result of genetic effects on different cell types. CRTh2 is expressed on airway smooth muscle and the peripheral nervous system (65). A direct role for CRTh2 in regulating asthma-related changes in lung function would be in line with a report of association between other CRTh2 polymorphisms and lower PC_20_ (26). *In silico* analysis of the CRTh2 rs533116 G > A locus suggests the A allele reduces binding of MEF2 [HaploReg, see text footnote 1; (63)], a transcription factor expressed by smooth muscle cells that regulates contraction (66). PGD_2_ activation of CRTh2 induces myocyte migration (9) and fibrosis (67) and so an enhanced CRTh2 pathway may increase airway smooth muscle area and tissue remodeling associated with asthma-related lower lung function (68). Observed differences in genotypic effects on type 2 inflammation (recessive) and FEV_1_ (dominant), may be due to chromatin accessibility at the CRTh2 locus differing within female and male cells and/or different cell types. Ultimately these nuclear environments may regulate the impact of CRTh2 rs533116 G > A.

Despite this novel finding of biological sex and age influencing the contribution of CRTh2 rs533116 G > A to asthma severity and type 2 inflammation, this study had some limitations. While data were acquired from two separate centers, it had to be pooled to achieve sufficient power to detect genotypic differences. We also lacked power to detect differences in some indices of symptom control, as number of exacerbations and cycles of oral corticosteroid/year were acquired only in a subset of subjects. Another important question is whether CRTh2 rs533116 G > A associates with a particular subtype of Type 2 high asthma. Previous cluster analysis identified two subtypes of female-dominant asthma characterized by moderate airflow obstruction (>68% predicted FEV_1_) and type 2 inflammation (either IgE or airway eosinophils) but differing in age-of-onset (< or >40 years of age) (69). Unfortunately, we could not assess the influence of CRTh2 rs533116 AA on age-of-onset or severity of asthma within each subtype due to low sample size (U of A, *n* = 59, AA < 40 = 4; AA > 40 = 2). Since the PGD_2_-CRTh2 pathway plays a role in both IgE-mediated and eosinophilic type 2 inflammation, CRTh2 rs533116AA may serve as a biomarker of severity for both early and late onset asthma. In light of these remaining questions, a follow up validation study including these outcomes and features of asthma is warranted and would extend our understanding of the current findings.

Severe asthma comprises about 5–10% of asthmatics, though this group accounts for more than 50% of the asthma related total health costs due to hospital admissions, use of emergency service and unscheduled physician visits (70–72). Our study reveals that CRTh2 rs533116 AA associates with having more type 2 inflammation, need for ICS and severe asthma diagnosis exclusively in females. As such, this variant may serve as a sex-specific biomarker for type 2 high severe asthma and help guide precision delivery of anti-Th2 therapy.

## Data availability statement

The raw data supporting the results and conclusions of this article will be made available by the corresponding author on reasonable request.

## Ethics statement

The studies involving human participants were reviewed and approved by University of Alberta Human Ethics Committee and Western University Human Ethics Committee. The patients/participants provided their written informed consent to participate in this study.

## Author contributions

NS acquired all flow cytometry and qRT-PCR data and performed genotyping of the U of A dataset and performed all statistical analyses. CM, CL, and RK oversaw recruitment of patients and acquisition of clinical data for the WU dataset. RK also oversaw genotyping of the WU dataset. HV oversaw recruitment and genotyping of all U of A patients and provided invaluable discussion for data interpretation and writing of the manuscript. LC conceived the project, coordinated the team, oversaw acquisition and analysis of the flow cytometry and qRT-PCR data, and wrote the final manuscript. All authors contributed to the article and approved the submitted version.

## Footnotes

1
https://pubs.broadinstitute.org/mammals/haploreg
