# Supplementary material for: The CRTh2 polymorphism rs533116 G > A associates with asthma severity in older females
Source: Front Med (Lausanne). 2022 Oct 13;9:970495. doi: 10.3389/fmed.2022.970495 (PMC9606418; doi:10.3389/fmed.2022.970495)
Supplement: Supplementary file 1 [file Data_Sheet_1.pdf]

Supplementary Material

The CRTh2 Polymorphism rs533116 G>A associates with Asthma Severity in Older Females

Nami Shrestha Palikhe[1], Constance A. Mackenzie[2,3,4], Christopher Licskai[2], Richard B. Kim[3], Harissios Vliagoftis[1] and Lisa Cameron[1,5]

[1]Division of Pulmonary Medicine, Department of Medicine and Alberta Respiratory Centre, University of Alberta, Edmonton, Alberta, Canada; [2]Division of Respirology and [3]Division Clinical Pharmacology, Department of Medicine, Western University, London, Ontario, Canada; [4]Ontario Poison Centre, Hospital for Sick Children, Division of Clinical Pharmacology and Toxicology, Toronto, Ontario, Canada; [5]Department of Pathology and Laboratory Medicine, Schulich School of Medicine & Dentistry, Western University, London, ON, Canada

***Correspondence:** Lisa Cameron, Department of Pathology Laboratory Medicine, Schulich School of Medicine and Dentistry, Western University, Dental Sciences Building, Rm 4037, London, ON, Canada N6A 5C1; Telephone: 519-661-2111-84305; FAX: 519-661-3370; Email: lisa.cameron@schulich.uwo.ca

Supplementary Tables

Supplementary Table S1. Clinical characteristics stratified by biological sex

Characteristics*	Females (n = 106)	Males (n = 64)	*p*
**Age**			
**All**	53.65 ± 1.51	48.82 ± 1.93	0.050
**≥ 45 years**	60.37 ± 0.98	58.90 ± 1.32	0.465
**BMI**			
**All**	31.26 ± 0.79	30.89 ± 0.86	0.747
**≥ 45 years**	31.65 ± 0.84	31.31 ± 0.92	
**FEV_1 (% predicted)**			
**All**	82.58 ± 1.96	74.75 ± 2.36	0.013
**≥ 45 years**	83.04 ± 2.00	74.46 ± 2.51	0.034
**FEV_1/FVC**			
**All**	68.79 ± 1.14	61.20 ± 1.57	0.000
**≥ 45 years**	68.31 ± 1.14	60.31 ± 1.66	0.001
**Total daily dose ICS***			
**All**	1076.95 ± 64.71	1122.21 ± 94.44	0.683
**≥ 45 years**	1063.86 ± 60.88	1203.08 ± 6.48	0.273
**Oral Corticosteroid****			
**All**	18.0 ± 4.0	32.0 ± 6.0	0.096
**≥ 45 years (%)**	17.0 ± 4.0	39.0 ± 6.0	0.014

*ICS: Inhaled Corticosteroid, budesonide equivalent;
**No difference in systemic steroid across genotypes;

Supplementary Table S2. Hardy Weinberg Equilibrium for CRTh2 rs533116 G>A

Genotype	Observed	Expected
**GG**	58	57.56
**GA**	83	83.88
**AA**	31	30.58
Exact test	0.8767	

Supplementary Table S3. Association of CRTh2 rs533116 G>A with severe asthma

	**Genotype**	**Total** n (%)	**Mild/ moderate** n (%)	**Severe** n (%)	**Dominant** GG vs GA/AA OR (95% CI)	***p***	**Recessive** GG/GA vs AA OR (95% CI)	***p***
**All***	GG	58 (34.1%)	32 (29.4%)	26 (34.1%)				
	GA	82 (48.2%)	60 (55.0%)	22 (36.1%)				
	AA	30 (17.6%)	17 (15.6%)	13 (21.3%)	0.76 (0.54 -1.06)	0.102	1.16 (0.78-1.77)	0.458
**Females****	GG	31 (29.2%)	18 (24.7%)	13 (39.4%)				
	GA	58 (54.7%)	46 (63.0%)	12 (36.4%)				
	AA	17 (16.0%)	9 (12.3%)	8 (24.2%)	0.67 (0.43 - 1.06)	0.085	1.48 (0.86-2.52)	0.155
**Males****	GG	27 (42.2%)	14 (38.9%)	13 (46.4%)				
	GA	24 (37.5%)	14 (38.9%)	10 (35.7%)				
	AA	13 (20.3%)	8 (22.2%)	5 (17.9%)	0.85 (0.52 - 1.41)	0.538	0.87 (0.46-1.63)	0.655

*Adjusted for age, sex and BMI; **Adjusted for age and BMI; OR, odds ratio; 95% CI, 95% confidence interval; *p*: Chi-Square

Supplementary Table S4. Association of CRTh2 rs533116 G>A with severe asthma in older subjects

**≥ 45 years**	**Genotype**	**Total** n (%)	**Mild/ moderate** n (%)	**Severe** n (%)	**Dominant** GG vs GA/AA OR (95% CI)	***p***	**Recessive** GG/GA vs AA OR (95% CI)	***p***
**Combined***	GG	44 (37.0%)	26 (33.3%)	18 (43.9%)				
	GA	56 (47.1%)	43 (55.1%)	13 (31.7%)				
	AA	19 (16.0%)	9 (11.5%)	10 (24.4%)	0.82 (0.55 - 1.21)	0.316	1.54 (0.93- 2.55)	0.093
**Females****	GG	27 (34.2%)	17 (30.4%)	10 (43.5%)				
	GA	41 (51.9%)	35 (62.5%)	6 (26.1%)				
	AA	11 (13.9%)	4 (7.1%)	7 (30.4%)	0.75 (0.46 - 1.24)	0.332	2.50 (1.26-4.98)	**0.009**
**Males****	GG	17 (43.5%)	9 (40.9%)	8 (47.1%)				
	GA	14 (35.9%)	8 (36.4%)	6 (35.3%)				
	AA	8 (20.5%)	5 (22.7%)	3 (17.6%)	0.87 (0.46 - 1.66)	0.686	0.862 (0.38-1.92)	0.717

*Adjusted for sex and BMI for combined; **adjusted for BMI; OR, Odd Ratio; 95% CI, 95% confidence interval
p: Chi-Square

Supplementary Table S5. Association of CRTh2 rs533116 G>A with FEV_1 (% predicted)

	**Genotype**			**Dominant**	**Recessive**	**Genotypic**
	GG	GA	AA	GG vs GA/AA *p*	GG/GA vs AA *p*	AA vs GG *p*
**All Asthmatics**	84.79±2.65	76.79±2.25	77.56±3.31	**0.017**	0.104	0.104
**≥ 45 years**	87.93±3.09	75.55±2.82	72.63±3.30	**<0.001**	**0.005**	**0.005**
**Females**	88.38±3.88	79.81±2.61	81.35±4.48	0.058	0.263	0.263
**≥ 45 years**	91.74±3.88	78.12±3.25	75.54±4.66	**0.004**	**0.023**	**0.023**
**Males**	80.66±3.47	69.48±3.97	72.61±4.74	**0.033**	0.187	0.187
**≥ 45 years**	81.88±4.91	68.53±5.42	68.62±4.45	**0.036**	0.104	0.104

Supplementary Table S6. Clinical characteristics of those examined for type 2 inflammation

**Characteristics**	**Mild/moderate** (N=35)	**Severe** (N=24)	***p***
**Age**	41.3 ± 2.45	42.9 ± 2.82	1.000
**Sex (% female)**	20 (57.1)	12 (50.0)	0.598
**BMI**	35.2 ± 2.83	30.71 ± 2.88	0.127
**FEV_1 (% predicted)**	85.4 ± 2.36	68.63 ± 4.05	**0.004**
**FVC (% predicted)**	98.7 ± 1.78	95.1 ± 4.25	0.938
**FEV_1/FVC**	85.5 ± 2.00	70.04 ±2.85	**< 0.001**
**Total daily ICS***	403.0 ±54.66	1141.0 ±49.30	**< 0.001**
**Smoking status**** No (%)	14 (40.0)	12 (50.0)	0.457

FEV_1: Forced expiratory volume in 1 sec; FVC: Forced vital capacity; ICS: Inhaled Corticosteroid, budesonide equivalent; ** Smoking status ever;

Supplementary Figure S1

A

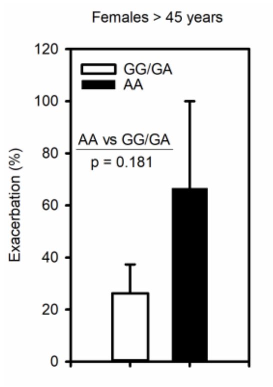

B

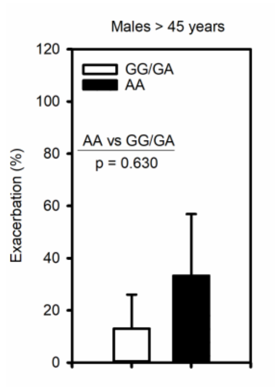

Supplementary Figure Legend

Figure S1. No association between the CRTh2 rs533116 G>A polymorphism and asthma exacerbation. Asthmatics were genotyped and assessed for the likelihood of having a serious exacerbation (emergency room visit or hospitalization). ***A)*** Older females (AA = 3, GG/GA = 13) and ***B)*** older males (AA = 3, GG/GA = 8) were assessed for the influence of the A allele on this outcome. Statistical differences determined Mann Whitney U-Test.
